# The Utility of Extracorporeal Membrane Oxygenation in the Setting of Chronic Thromboembolic Pulmonary Hypertension

**DOI:** 10.3390/medsci14020273

**Published:** 2026-05-28

**Authors:** Ayman Mohammed, Saada Hussein, Ghadeer Mahdi, Amir Hossein Behnoush, Robert D. Schultz, Marco Tagliafierro, Ian Mason, Yoshiko Ishisaka Mori, Toshiki Kuno, Kaveh Hosseini, Ali Fatehi Hassanabad

**Affiliations:** 1 Section of Cardiac Surgery, Department of Cardiac Sciences, Libin Cardiovascular Institute, Cumming School of Medicine, University of Calgary, Calgary, AB T2N 4N1, Canada; 2 Cardiovascular Imaging Core Laboratory, Northwestern University Feinberg School of Medicine, Chicago, IL 60611, USA; 3 Section of Cardiac Surgery, Department of Surgery, Columbia University Medical Center, New York Presbyterian University, New York, NY 10032, USA; 4 Division of Pulmonary, Critical Care, and Sleep Medicine, Northwell Health, Donald and Barbara Zucker School of Medicine at Hofstra/Northwell, New Hyde Park, NY 11040, USA; 5 Division of Cardiology, Beth Israel Deaconess Medical Center, Harvard Medical School, Boston, MA 02215, USA; 6 Department of Cardiology, Copenhagen University Hospital-Herlev and Gentofte, DK-2900 Copenhagen, Denmark

**Keywords:** mechanical circulatory support, chronic thromboembolic pulmonary hypertension, clinical outcomes, ventricular dysfunction, pulmonary hypertension

## Abstract

Chronic thromboembolic pulmonary hypertension (CTEPH) is a progressive disease that occurs due to fibrotic remodeling of the pulmonary vessels. This leads to increased pressure overload onto the right ventricle, resulting in complications such as heart failure. Pulmonary endarterectomy (PEA) remains the gold standard of treatment for CTEPH, yet many patients experience life-threatening perioperative complications, including refractory right ventricular failure, reperfusion pulmonary edema, and endobronchial hemorrhage. Extracorporeal membrane oxygenation (ECMO) has been used as a form of mechanical circulatory support to aid recovery in patients with perioperative complications in the context of CTEPH. This review identifies preoperative risk factors, including pulmonary vascular resistance, high body mass index, and elevated neutrophil-to-lymphocyte ratios. It also identifies differences in ECMO configuration, with veno-arterial ECMO preferred for hemodynamic instability and veno-venous ECMO for respiratory failure. Finally, we posit that, based on contemporary literature, the implementation of early ECMO in decompensated patients may be associated with reduced hospital mortality, and in those who survive beget excellent mid-term survival.

## 1. Introduction

**Figure 1:**
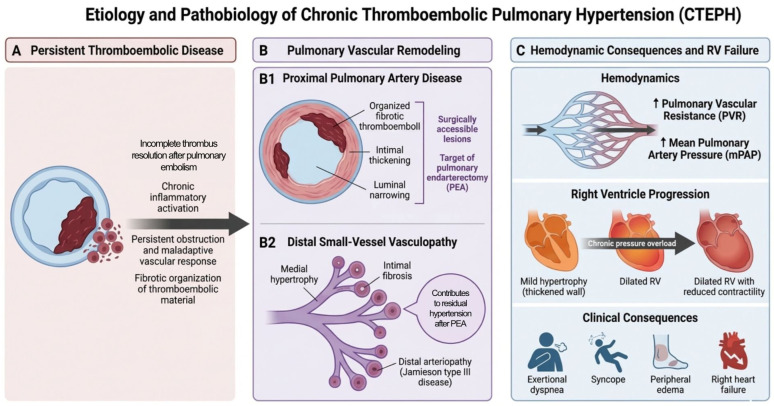
CTEPH develops from persistent organized thromboembolic obstruction and secondary small-vessel vasculopathy, resulting in elevated pulmonary vascular resistance, pulmonary hypertension, and progressive right ventricular failure.

Chronic thromboembolic pulmonary hypertension (CTEPH) is a critical, progressive, and life-threatening condition that develops as a complication of thrombo-emboli induced remodeling of the pulmonary vasculature and subsequent pulmonary hypertension. The pathophysiology of CTEPH involves the incomplete resolution of thrombo-emboli within the pulmonary vasculature, leading to organized fibrotic remodeling (Figure 1). This obstruction increases pulmonary vascular resistance (PVR) and results in right ventricular pressure overload. Over time, this process can progress to right heart failure in patients with untreated CTEPH. Currently, CTEPH is clinically defined by a mean pulmonary artery pressure (mPAP) greater than 20 mmHg, a pulmonary artery wedge pressure (PAWP) of 15 mmHg or less, and a PVR greater than 2 Wood units [1]. Of note, many of the studies cited in this review predate the 2022 ESC/ERS guidelines, which lowered the diagnostic threshold for pulmonary hypertension from a mPAP ≥ 25 mmHg to >20 mmHg.

For patients with surgically accessible disease, pulmonary endarterectomy (PEA) remains the gold standard treatment in resolving organized thrombo-emboli. With appropriate surgical intervention and careful pre- and post-operative management, PEA can reduce operative mortality rates to less than 5% [2]. While PEA is an extremely effective tool in the treatment of CTEPH, there can still be severe perioperative complications such as right ventricular failure, reperfusion pulmonary edema, residual pulmonary hypertension, secondary left heart failure, surgical complications including hemorrhage (both endobronchial and pulmonary), difficulty weaning from cardiopulmonary bypass (CPB), and infection.

**Figure 2:**
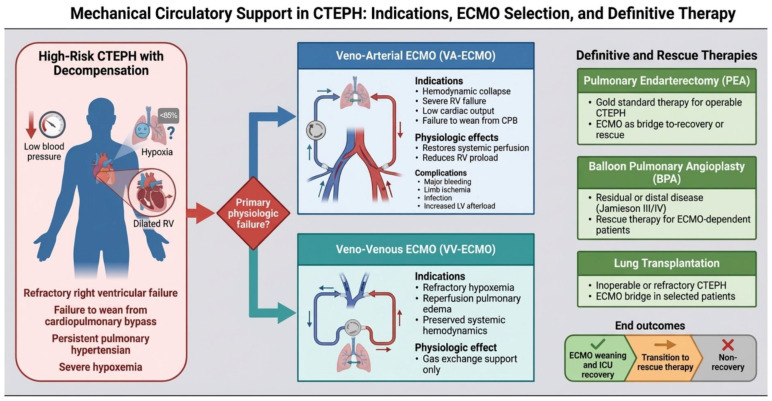
ECMO provides perioperative stabilization, rescue support, and bridging to definitive or rescue therapies in high-risk CTEPH, with modality selection guided by hemodynamic versus respiratory failure.

Mechanical circulatory support (MCS) in the form of extracorporeal membrane oxygenation (ECMO) has emerged as an important adjunct in the management of severe perioperative complications in CTEPH. The primary indications for ECMO in this setting are severe pulmonary hypertension and right heart failure, with multiple secondary indications that include refractory hypoxemia from reperfusion pulmonary edema, endobronchial bleeding, and the inability to wean from bypass (Figure 2). Therefore, ECMO can act as a crucial salvage therapy in managing cardiorespiratory failure. There are two types of ECMO: veno-venous ECMO (VV ECMO) and veno-arterial ECMO (VA ECMO) [3]. VV ECMO is commonly employed to reduce lung burden, often in the context of refractory hypercapnic respiratory failure, refractory hypoxemia, or as a bridge to lung transplants. In contrast, VA ECMO provides combined cardiac and respiratory support by oxygenating drained venous blood prior to returning it into systemic arterial circulation, thereby decompressing pulmonary circulation and effectively reducing right ventricular workload. This is commonly utilized in the setting of cardiogenic shock, low cardiac output, post-operative heart failure, and other similar conditions [4]. A peripheral or central cannula can be used to collect blood from the right atrium and deliver it to peripheral arteries. Hybrid configurations such as veno-arteriovenous (V-AV) or veno-venoarterial (V-VA) ECMO may be considered in select scenarios requiring combined support. These hybrid strategies have cannulation to both venous and arterial systems [5].

In this narrative review, we summarize the evidence supporting the use of ECMO in the management of high-risk operative CTEPH. We offer indications for the timing of instituting ECMO, including preoperative bridging, intraoperative transition, and postoperative rescue. We also establish preoperative risk stratification and evaluate MCS configurations. In doing so, we critically evaluate outcomes, including mortality and rate of complications.

## 2. Methods

This narrative review was conducted by synthesizing available evidence on the use of ECMO in the management of CTEPH. A structured literature search was performed using PubMed and Scopus databases from database inception to 30 January 2025. Search terms included combinations of “CTEPH,” “extracorporeal membrane oxygenation,” “ECMO,” “mechanical circulatory support,” “pulmonary endarterectomy,” “balloon pulmonary angioplasty,” and related terms, including their full-text equivalents. Articles where limited to those published in the English language. The search yielded 20 records, all of which underwent full text review. 9 were included in the final synthesis. All screening and study selection was done by two authors (AM, AFH). We included a range of study designs, including case reports, case series, observational studies, and relevant meta-analyses. Studies were selected based on their relevance to the use of ECMO in the perioperative and critical care management of CTEPH. The identified studies were qualitatively synthesized, focusing on key domains including preoperative risk stratification, indications and timing of ECMO initiation, configuration strategies, clinical outcomes, and associated complications. Table 1 presents the included studies and the key findings including the study design, the sample size, key outcomes and the 95% confidence interval when available. A qualitative risk of bias assessment was performed by the authors. Instead of a formal scoring tool, we opted to assess bias based on study design, sample size, selection bias, outcome reporting and generalizability. Studies were then classified as low, moderate, or high risk. Case reports were used in this review to demonstrate mechanisms or strategies rather than suggest treatment effects and were classified in our table to be ‘high risk’.

## 3. Preoperative Risk Stratification and Patient Selection

The accurate identification of high-risk patients and an understanding of the advanced mechanical circulatory supports are critical to improving patient outcomes before, during, and after PEA. Evidence indicates that anatomical, hemodynamic, and clinical factors are strongly associated with increased perioperative risk in patients with PEA. These factors help identify patients at increased risk of perioperative complications and those who may benefit from early consideration of mechanical circulatory support.

### 3.1. Hemodynamic Instability

Hemodynamic parameters are among the most consistent predictors of perioperative risk. Elevated pulmonary vascular resistance (PVR) has been identified as a key determinant of perioperative ECMO requirement across multiple studies [6,7]. Wang et al. utilized machine learning models to identify PVR as a primary characteristic predictor of ECMO demand (area under the curve [AUC]: 0.847, 95% CI 0.752–0.942) and found that the optimal threshold for predicting ECMO need was 1070.5 dyn·s/cm^5^ (~13.4 Woods units) [7]. Likewise, they identified that low flow states, characterized by a cardiac index lower than 1.8 L/min/m^2^ and a cardiac output lower than 3.3 L/min, were significant indicators that a patient may require ECMO for hemodynamic support [7]. These estimates were derived from a single center cohort of 117 patients, of whom only 8 received ECMO. Therefore, the reported AUC threshold and low flow state values require external validation before clinical application. Structural markers of advanced disease, particularly severe right ventricular dilation, were also associated with the requirement of mechanical support (discussed further in Section 3.4) [6]. Taken together, these findings suggest that a combination of markedly elevated PVR, reduced cardiac output, and evidence of right ventricular remodeling identifies a high-risk hemodynamic phenotype that may benefit from early consideration of ECMO support in the perioperative setting.

### 3.2. Surgical and Anatomical Classification

While PEA requires surgical skills and technique, proximal thrombo-emboli are more easily accessible. The Jamieson disease classification, originally defined by Jamieson and colleagues in their landmark series of 150 PEA operations, created a model to define the location of these thromboembolic events [8]. These classifications were validated by Thistlethwaite and colleagues [9]. Type I is classified by fresh thrombus in the main and lobar pulmonary arteries, type II is defined by intimal thickening with fibrosis proximal to the segmental arteries, type III is defined as fibrosis and intimal webbing confined to the segmental arteries, and type IV is characterized by pure small vessel vasculopathy [9]. Type III and IV occlusions, due to distal small-vessel vasculopathy, represent a major predictor of mortality and weaning failure, as these lesions are often surgically inaccessible [10,11,12]. Even a technically perfect PEA may still result in persistent or residual mPAP > 30 mmHg. Abdelnour-Berchtold et al. noted that 66% of their patients with decompensated right heart failure (DRHF) requiring VA ECMO had type III disease [13].

While the original Jamieson disease classification from the University of California San Diego (UCSD) helped define pulmonary artery occlusion, they adopted a new scale based on location and surgical findings [10]. Madani and colleagues introduced a level based scheme, now referred to as the UCSD classification, which approaches disease classification by anatomical location rather than morphology as observed in the Jamieson classification. They classify disease by where fibrotic material is first encountered at endarterectomy. Level 0 denotes no disease; Level 1 is obstruction at one of the main pulmonary arteries, with 1C classification added for complete single lung occlusion. Level 2 begins at the lobar branches, level 3 at the segmental branches, and level 4 at the subsegmental branches [10]. Levels 3 and 4 pose the greatest technical challenge for PEA. These evolving classification systems further emphasize the importance of anatomical assessment in predicting perioperative risk and guiding management strategies.

### 3.3. Biomarkers

Emerging evidence suggests systemic inflammatory and cardiac markers can predict postoperative complications that may prompt ECMO use [7]. In the same Wang et al. cohort, the preoperative neutrophil-to-lymphocyte (N/L) ratio predicted ECMO therapy. Patients with an N/L ratio greater than 2.5 were shown to be likely to receive ECMO treatment, with an AUC of 0.896 (95% CI 0.803–0.989). Likewise, preoperative brain natriuretic peptide (BNP) and C-reactive protein (CRP) were higher in patients who eventually required mechanical support [7]. While these lab values can be correlated with the need for MCS, more data is needed to demonstrate a causal link between N/L, BNP, and CRP and perioperative ECMO deployment. These markers should be interpreted as adjunctive tools for risk stratification rather than definitive predictors, and further studies are needed to clarify their causal and prognostic roles. Their relevance is biologically plausible given that chronic inflammation and dysregulation of cytokine signaling molecules are correlated with pulmonary vascular remodeling in CTEPH [14].

### 3.4. Imaging Phenotypes

Several imaging modalities can be utilized to diagnose CTEPH and localize foci of chronic thrombo-emboli, including echocardiography, SPECT ventilation-perfusion (VQ), and CT pulmonary angiography (CTPA). Generally, echocardiography is the first step in diagnosing pulmonary hypertension in the setting of CTEPH and in stratifying patients. Several key echocardiographic parameters may differ for risk stratification between pulmonary arterial hypertension (PAH) and CTEPH. Markers of RV pressure overload such as peak tricuspid regurgitation (TR), flattening of the interventricular septum (left ventricular eccentricity index > 1.1 in systole and/or diastole), and right ventricular outflow acceleration time < 105 ms may better stratify risk in CTEPH than in PAH [15]. Further, evidence predicting the need for MCS emphasizes right ventricular dilation; preoperative echocardiographic evidence of an RV diastolic diameter greater than approximately 57 mm is an anatomical risk factor requiring ECMO support following PEA [16]. For CTEPH screening, SPECT VQ scintigraphy is the preferred imaging method. Utilizing a threshold of 2.5 segmental mismatched perfusion defects, a sensitivity of 100% and a specificity of 94.7% can be achieved [16].

Compared with CTPA, VQ scintigraphy has been demonstrated to have a higher sensitivity [17]. Although no direct studies demonstrating VQ-mismatch found on scintigraphy and peri-operative MCS have been performed, studies aimed at predicting recurrent pulmonary hypertension following PEA elucidate the potential prognostic implications of this imaging modality. Used in conjunction with a right-heart catheterization-derived mean-pulmonary arterial pressure cut-off of ≥44.5 mmHg, patients with a Ventilation-Perfusion Mismatch Defect (VPMD) above 35.58% had a higher likelihood of persistent/recurrent pulmonary hypertension and lower survival [18]. This study, which did not provide specific reference to MCS following intervention, demonstrated a subset of patients at higher risk for poor outcomes and thus warrants close observation for potential postoperative support.

Despite VQ scans providing higher sensitivity than CTPA, patient access to nuclear medicine imaging can be variable which can delay diagnosis and evaluation [19]. Similarly to VQ scintigraphy, no studies correlating perioperative MCS with preoperative CTPA exist. However, the literature demonstrates that patients at risk for persistent PH following PEA warrant close observation for potential postoperative support. Features on CTPA demonstrating potential for poor outcome include absence of central thromboembolic disease, number of abnormally perfused lobes (a higher number correlating with higher postoperative PVR), number of peripheral densities, and absence of bronchial artery dilatation [20].

### 3.5. Clinical Phenotypes

Clinical phenotypes, specifically body mass index (BMI), significantly influence surgical morbidity, a concept that is explored in numerous papers. In a retrospective analysis of 110 PEA patients requiring perioperative ECMO, researchers identified high BMI as an independent risk factor for worse outcomes, specifically in the subgroup of 39 patients who required ECMO for reperfusion pulmonary edema post PEA [11]. Non-survivors in this group had a mean BMI of 37.7 ± 10.0 kg/m^2^ compared to 28.1 ± 6.7 kg/m^2^ in survivors [11]. These findings suggest that increased BMI may contribute to adverse outcomes through mechanisms such as reduced pulmonary reserve, impaired respiratory mechanics, and increased susceptibility to secondary infections [11].

## 4. ECMO Configurations and Management

Application of ECMO in the context of CTEPH requires a detailed consideration of hemodynamics, anatomical considerations, and the intended clinical trajectory. Here, we discuss indications for ECMO, the choice of modalities, cannulation strategies, optimization, and complications.

### 4.1. Indications for ECMO

ECMO can be initiated for a variety of clinical scenarios in CTEPH management and can be deployed perioperatively in the setting of PEA. The most common indication for ECMO is failure to wean from CPB following a PEA procedure. Patients can present post-procedurally with right ventricular failure, refractory hypoxemia, or persistent pulmonary hypertension. These complications limit the patient’s ability to be removed from CPB and necessitate mechanical circulatory support. ECMO is therefore used as a direct transition from CPB to provide cardiorespiratory support [13]. ECMO can also be used as a bridge to PEA. Acute exacerbation of CTEPH can lead to cardiogenic shock or respiratory failure; ECMO can stabilize these conditions and help maintain stability for surgical intervention [21]. In cases of advanced-stage CTEPH in patients ineligible for PEA, lung transplant can be considered. In this setting, ECMO is used perioperatively during the transplant procedure to maintain hemodynamic stability [21]. Across all of these indications, the decision to deploy ECMO should be made with a multidisciplinary CTEPH team. The 2022 ESC/ERS guidelines give a Class I recommendation that every CTEPH patient be evaluated with a team composed of PEA-trained surgeons, pulmonary hypertension specialists, interventional cardiologists familiar with BPA, cardiac anesthesiologists and ECMO-capable ICU intensivists. While this review focuses on MCS, it is worth mentioning targeted medical therapy that should also be considered as part of the preoperative optimization. The guidelines give riociguat (soluble guanylate cyclase stimulator) a Class I recommendation for inoperable CTEPH and for persistent or recurrent pulmonary hypertension after PEA. Similarly, treprostinil (synthetic prostacyclin analogue) carries a Class IIb recommendation in the guidelines for this population cohort [1].

### 4.2. ECMO Modality

The selection between VA- and VV ECMO is guided by the underlying physiological deficit, specifically whether circulatory, respiratory, or combined support is required. VA ECMO is the modality of choice for patients with hemodynamic collapse, RV failure, failure to wean from CPB, and persistent pulmonary hypertension despite interventions. This is because cardiac output and systemic perfusion are inadequate for systemic circulation. By using VA ECMO, providers can increase mean arterial pressure and restore organ perfusion independently from native cardiac output. Additionally, VA ECMO reduces right-sided preload, making it a useful clinical tool in the setting of RV failure. In contrast, VV ECMO is used to provide respiratory support following severe respiratory failure, acute pneumonia, pulmonary edema, and other similar diseases. VV ECMO drains venous blood and returns it into the venous circulation; the heart still provides systemic blood flow. VV ECMO may also contribute to a reduction in PVR by reversing hypoxic pulmonary vasoconstriction (via perfusion of the pulmonary circuit with oxygenated blood) and through the vasodilatory effect of the relative hypocapnia. This modality can be particularly useful in those patients whose hemodynamic instability is driven by hypoxia-mediated PVR elevation, as VV ECMO corrects hypoxemia and hypercapnia by shunting blood through the external oxygenator [12,13].

Although both modalities are perioperatively used in the context of CTEPH and PEA, a retrospective analysis by Bertazzo and colleagues reported different mortality rates between patients requiring VA ECMO and those supported with VV ECMO post-PEA: all 4 patients on VA ECMO died, compared to 1 of 7 patients on VV ECMO [6]. This difference can likely be attributed to the severity of illness in patients who require VA ECMO rather than the superiority of one ECMO modality over another. Given the small denominators, no comparisons can be drawn from these findings, and larger studies are needed to establish differences in outcomes between ECMO modalities.

Grate and colleagues reported a patient with CTEPH placed on VA ECMO preoperatively as a bridge to surgery, but was transitioned to VV ECMO on day 28 due to refractory hypoxemia; the patient then underwent PEA on day 40.21 Beyond standard VA and VV setups, there also exist hybrid strategies such as veno-arteriovenous (V-AV) or veno-venoarterial (V-VA) ECMO. These hybrid strategies may help relieve burden, balance circulatory systems, reduce RV preload, and combat shortcomings. The hybrid configurations have been reported in the context of CTEPH for post-procedural patients, where neither respiratory nor circulatory support alone was adequate [6].

### 4.3. Cannulation Strategies

Central and peripheral cannulation can be employed to institute MCS in the setting of CTEPH and PEA. Each strategy poses advantages and disadvantages. Central cannulation with VA ECMO is used most commonly intraoperatively in the setting of an existing sternotomy and is frequently employed in patients who fail to wean from CPB due to persistent pulmonary hypertension, right ventricular failure, or severe reperfusion injury [12,22,23]. This approach also allows for high-flow circulatory support and has been associated with improved survival in high-risk patients, such as those with DRHF [13]. Several observational series have reported on the successful rate of ECMO weaning with centrally cannulated VA ECMO [12]. Peripheral cannulation may be used when no sternotomy is performed, or the chest incision needs to be closed, or when ECMO is initiated in the postoperative period. Peripheral ECMO has been widely utilized in the context of PEA for cardiopulmonary collapse or refractory hypoxemia and allows for rapid use without the need for surgical re-entry [7]. However, peripheral cannulation is associated with risks of limb ischemia and vascular injury and may have suboptimal loading in the setting of right ventricular failure. Central cannulation offers superior hemodynamic stability and lowers risks of peripheral vascular injury, but it necessitates re-sternotomy for decannulation and requires ongoing open-chest management [23]. Not specific to CTEPH, Totapally et al. found that peripheral cannulation was associated with a higher mortality rate compared to central cannulation in a pediatric population with CHF [24].

Beyond standard central and peripheral cannulation strategies, a percutaneous dual lumen cannula such as the ProtekDuo (LivaNova, London, UK) can be used. It is inserted into the right internal jugular vein, where one lumen drains blood from the right atrium, while the other returns oxygenated blood to the pulmonary artery. This creates a RV bypass circuit that allows for integrated oxygenation and selective unloading of the RV without affecting LV afterload. The evidence for this dual lumen strategy has not been well established in CTEPH but the configuration does align with the goals of MCS.

### 4.4. Hemodynamics Optimization and LV Unloading

One of the most concerning aspects of VA ECMO support for CTEPH management is the potential for chronic underfilling of the LV, combined with increased afterload. This happens as a consequence of retrograde backflow from the femoral artery cannula against which the LV must eject. This thereby increases afterload and precipitates secondary LV dysfunction. The result is increased LV end-diastolic pressure, potentially leading to pulmonary edema, particularly in the context of impaired right ventricular recovery [12]. This can be mitigated by titrating ECMO flow to balance systemic perfusion with LV unloading, often in conjunction with inotropes and vasoactive drugs to support cardiac function and myocardial contractility [12]. Some institutions have also used adjunctive pulmonary vasodilator therapy, such as inhaled nitric oxide or intravenous prostacyclin analogues, during the perioperative period to reduce RV afterload and decrease PVR [7]. These drugs can reduce RV strain, consequently decreasing PVR and increasing LV preload. Other adjunctive interventions can be considered, including atrial septostomy to decompress the right heart [25]. An alternative strategy for mechanical LV unloading during VA-ECMO is the ECpella configuration, in which an axial-flow Impella device (Abiomed, Danvers, MA, USA) is used to decompress the LV. This is done by aspirating blood from the LV cavity and transferring it into the ascending aorta while ECMO continues to provide systemic perfusion and gas exchange. The evidence of this setup in CTEPH is limited and requires further exploration.

## 5. Outcomes

Although ECMO can be an intervention that saves lives, it presents substantial risks that can manifest in the context of high-risk post-PEA procedures. This risk is amplified in those with significant baseline comorbidities, including elevated PVR, right ventricular dysfunction, or obesity. Successful outcomes are dependent on proactive management, choosing the appropriate patients, and navigating complications.

### 5.1. Mortality and Survival

The use of ECMO as a salvage therapy is underscored by high inpatient mortality rates. In a meta-analysis, Ishisaka et al., found a mortality rate of 43.5% for CTEPH patients requiring perioperative ECMO [12]. Similar results were reported in individual cohort studies, with ECMO group mortality ranging from 37.5% (3 of 8 patients) to 45.5% (5 of 11 patients) [6,7]. In contrast, Abdelnour-Berchtold et al. reported that the introduction of a proactive strategy of central VA ECMO bridge to recovery in patients with preoperative DRHF prior to going for PEA coincided with a reduction in mortality for that cohort from 31% (between 2005 and 2013) to 4% (2014–2019); of note, this group defined the DRHF cohort as patients with fluid overload, low CO, and secondary organ failure [13]. Between 2014 and 2019, the team saw 29% of the DRHF patients transitioned intraoperatively from CPB to VA ECMO. A parallel reduction in mortality was observed in patients with a preoperative total pulmonary resistance (TPR, noted as mPAP/CO, which differs from PVR by not subtracting the PAWP) greater than 1200 dyn·s/cm^5^, from 13.2% to 1.7% across the same time frames. However, it is important to note that this was a single center before-and-after study, and the improvement cannot be attributed to ECMO use alone; this must be understood in the context of evolving surgical technique, postoperative management and patient selection. These findings do still suggest that using ECMO as a preemptive tool in well-defined high-risk populations may be associated with better outcomes. More large-scale research is needed to explore outcomes in VV ECMO groups as well.

### 5.2. Bleeding Outcomes

Bleeding represents a significant life-threatening challenge for patients with CTEPH requiring ECMO. One study noted that in high-risk cohorts requiring ECMO following PEA, the rate of life-threatening airway bleeding has been reported at 36.3%, compared to 3.2% in those who did not require ECMO [6]. A broader systematic review suggest that bleeding complications in perioperative PEA ECMO can be anywhere from 12.2% to 22.0% [12]. These populations require increased transfusion rates and can be challenging to manage from both a surgical and a cost perspective, given the limited availability of blood, platelet, and fresh frozen plasma products. Surprisingly, one study found that the outcomes for ECMO patients with endobronchial bleeding are favorable, reporting a 74.2% salvage rate. This salvage rate refers to the successful weaning of patients from ECMO support who were established on the circuit due to life-threatening endobronchial bleeding [11]. This rate of success can be attributed to the use of heparin-coated circuits, which may allow clinicians to safely reverse anticoagulation with agents such as protamine while the lungs are relaxed. However, heparin-coated circuits alone are often insufficient, particularly in CTEPH patients as they often have abnormal coagulation systems, including thrombophilia and antiphospholipid syndrome [6]. In such cases it is important to consider the use of tools such as the Heparin Management System (HMS, Medtronic, Minneapolis, MN, USA) to detect heparin resistance and individualize dosing. For longer ECMO runs, one can consider direct thrombin inhibitors including bivalirudin as an alternative to unfractionated heparin.

However, while these successes highlight the changing nature of bleeding risk in ECMO, it is important to note the poor outcomes, refractory coagulopathy, and increased mortality. In that same cohort, those with reperfusion pulmonary edema had a weaning survival rate of 56.4%, those with RV failure had a 40% weaning survival rate, and the total cohort of all 110 ECMO patients had a 56.4% weaning survival rate [11]. It is also important to distinguish between successful weaning and survival to discharge; while 74.2% (23/31) of patients in the endobronchial bleeding cohort were successfully weaned from ECMO support, 71.0% (22/31) of the entire subgroup survived to be discharged alive from the hospital [11]. These findings underscore the complexity of bleeding management in ECMO-supported patients and highlight the distinction between ECMO weaning success and overall survival.

### 5.3. Infectious Complications

Prolongation of ECMO support renders patients highly susceptible to severe nosocomial infections. Grate et al. reported that the diagnosis of nosocomial infections in this population is confounded by a systemic inflammatory response induced by the ECMO circuit itself [21]. This leads to the masking of the pyrexia caused by extracorporeal temperature regulation. This means that bloodstream infections leading to septic shock and contamination of ECMO machines can go unnoticed. In this case, Klebsiella bacteremia led to the formation of a purulent thrombus within the oxygenator. The infected thromboembolic material led to inoculation of the patient’s pulmonary arteries and acted as the septic source [21]. Antibiotic management was insufficient to contain the infection, and the patient underwent a PEA for surgical source control. This is an important complication to consider when thinking about ECMO for CTEPH patients. Reports suggest that nosocomial infection rates are as high as 20.5%, with pooled sepsis rates noted at 8.8% in perioperative ECMO use for PEA [12,21]. In a single center series, Bertazzo et al. similarly reported that 7 of their 11 patients (63.6%) requiring ECMO after thrombectomy had their course complicated by sepsis.

## 6. Alternative and Rescue Pathways

Failure to wean from ECMO can be challenging from an interventional standpoint. The team may consider advanced interventions that may be necessary to prevent mortality. For these patients, the focus shifts to rescue therapy.

### 6.1. Rescue Balloon Pulmonary Angioplasty

Balloon pulmonary angioplasty (BPA) has evolved into a recognized treatment modality in CTEPH. Current 2022 ESC/ERS guidelines recommend BPA as a class I therapy for patients with inoperable disease and as an adjunct for persistent pulmonary hypertension after PEA [1]. In the case of ECMO patients, BPA additionally serves as a rescue intervention for those who remain ECMO-dependent post-PEA. BPA is indicated for those with Jamieson Type III/IV distal disease that is surgically inaccessible during PEA, as it can target segmental and subsegmental branches where small vessel vasculopathy prevents immediate reduction in pulmonary pressure. Nakamura and colleagues described a case in which they deployed BPA in a patient who could not be weaned from VA ECMO post-PEA. BPA was used as a tool to target the remaining peripheral stenotic lesions, which subsequently improved pulmonary hemodynamics and pulmonary pressures leading to patients’ weaning from ECMO [26]. A similar strategy was employed by Sugiyama et al., who showed that post-operative BPA is required in up to 75% of ECMO survivors [23].

### 6.2. Lung Transplant

Complications in CTEPH patients who fail to have symptom resolution after PEA can be considered for lung transplant. While PEA can be curative, up to 40% of CTEPH patients are deemed inoperable due to the distal location of the vessel disease. A case series by Chen et al. reported the outcomes observed with this approach. Seven patients were followed, all of whom underwent lung transplants for CTEPH. All seven survived to discharge, with significant improvement in mPAP. One of these patients had persistent oxygenation failure and was successfully supported on preoperative ECMO for 32 days before undergoing bilateral lung transplantation. Modality of ECMO matters; three of the seven patients were placed on VV ECMO, and the remainder on VA ECMO. If the patient’s hemodynamics are stable, VV ECMO is the preferred modality. Alternatively, a drop in oxygen saturation below 90% may warrant a transition to VA ECMO [22].

## 7. Perspective and Future Directions

### 7.1. Limitations of the Current Evidence

The current body of literature on the evidence of ECMO use in CTEPH is constrained by several limitations. First, most available data are derived from case reports, small case series, and single-center retrospective studies, which are inherently prone to selection bias and limit the generalizability of findings. No prospective or randomized data exists to guide ECMO use in the CTEPH population. Second, significant heterogeneity in institutional protocols for ECMO initiation and management strategies cannot be excluded, making direct comparisons between population cohorts challenging and limiting the extent to which findings can be standardized across different population groups. In addition, those patients placed on ECMO are likely those that will deteriorate or fail to wean from bypass, thus making comparisons between non-ECMO and ECMO groups challenging. Reported mortality in ECMO groups often reflects the severity of the underlying physiology rather than the mechanical support itself. Third, most reports do not stratify outcomes based on Jamieson or UCSD anatomical classification, ECMO timing or ECMO indication; this makes it difficult to quantitatively assess the survival benefit attributable to ECMO.

### 7.2. Future Directions

The preemptive use of ECMO in CTEPH is not universally possible, and more studies are needed to help define the patient populations that benefit the most from ECMO pending PEA. One example is PVR; while elevated PVR has consistently emerged as a key predictor of ECMO requirement, additional clinical, imaging, and biomarker-based variables may further refine risk stratification. Future studies should aim to develop and validate comprehensive predictive models incorporating hemodynamic parameters, inflammatory markers such as the neutrophil-to-lymphocyte ratio, and imaging-derived metrics to improve patient selection for ECMO.

In addition, the role of BPA, both as a primary therapy for inoperable CTEPH and as a rescue intervention in ECMO-supported patients, requires further investigation to define optimal timing, patient selection and integration with other strategies. Clarifying whether these interventions improve short- and long-term outcomes in ECMO-supported patients will be critical. Overall, prospective, multicenter studies are needed to establish standardized protocols for ECMO utilization, optimize timing of initiation, and improve outcome prediction in this high-risk population. Finally, the utility of other MCS devices, including intra-aortic balloon pumps, micro-axial pumps (Impella 5.5, Abiomed, Danvers, MA, USA), and right ventricular support devices (Impella RP, Abiomed, Danvers, MA, USA) in managing patients with hemodynamic collapse or right ventricular failure secondary to CTEPH should be thoroughly and critically evaluated in large, multicenter studies. In particular, left-sided micro-axial pumps may exacerbate RV failure by increasing pulmonary blood flow against an obstructed pulmonary bed. Additionally, an RV support device could drive flow into that obstruction and lead to higher pulmonary pressure and possible hemorrhage. The role of these devices in CTEPH should be investigated cautiously.

## 8. Conclusions

MCS, particularly ECMO, is becoming a vital component in the management of CTEPH. Historically, it has been viewed as a safety net for specialized surgical centers or as a bridge to or from PEA. However, the integration of ECMO into perioperative protocols has expanded surgical eligibility by allowing for the successful management of patients with extreme hemodynamic impairment and complicated thromboembolic events. Emerging evidence suggests that a proactive approach with central VA ECMO in certain contexts, such as decompensated RV function, may be associated with improved survival and functional recovery. Despite the high incidence of complications, including infection and bleeding, ECMO provides the critical physiological unloading of the right ventricle to allow it to recover in the setting of PEA. MCS also enables alternative rescue pathways, including BPA and lung transplants, for those patients whose conditions do not resolve with PEA and ECMO support. Further advances in CTEPH care will rely on the continued refinement of existing protocols and validation of novel biomarkers to individualize mechanical support strategies.

## Figures and Tables

**Table 1:** Included studies of ECMO and other MCS in PEA for CTEPH.

Study	Design	Sample Size	Primary Outcomes and 95% Confidence Intervals	Risk of Bias
Ishisaka et al., 2023	Systematic review and meta-analysis.	11 studies; 2632 PEA patients.	ECMO insertion rate was 8.7% (95% CI 5.9–12.5). Mortality was 43.5% (30.8–56.2) in ECMO patients vs. 2.8% (1.7–4.5) without ECMO. The weaning rate was 72.6% (53.4–91.7).	Moderate to high. Pools observational studies that varied a lot in how ECMO was used.
Chia et al., 2024	Retrospective cohort from the UK national PEA center.	110 ECMO cases (4.7% of the PEA cohort).	62 of 110 patients were weaned (56.4%; 46.6–65.8) and 57 were discharged alive (51.8%; 42.1–61.4). Distal disease and residual PH were linked to higher mortality.	Moderate. Large cohort but retrospective.
Abdelnour-Berchtold et al., 2022	Single-center before/after cohort.	388 PEA patients, 40 with decompensated RHF, 13 central VA-ECMO cases.	After central VA-ECMO was introduced, mortality in the RHF group dropped from 31% to 4% (*p* = 0.03). 12 of 13 patients on central VA-ECMO survived to discharge (92.3%; 64.0–99.8).	High. A before/after design cannot separate the effect of VA-ECMO from other changes over time.
Wang et al., 2022	Single-center retrospective cohort with predictive modeling.	117 PEA patients, 8 ECMO cases.	PVR and the neutrophil-to-lymphocyte ratio predicted ECMO use, with AUCs of 0.85 and 0.90. 3 of 8 patients died and 6 of 8 were weaned.	High. Only 8 ECMO events and no external validation.
Bertazzo et al., 2024	Single-center retrospective cohort.	42 PTE patients, 11 ECMO cases.	ECMO was used in 26.2% of patients. Mortality was 45.5% in the ECMO group, with all 4 VA-ECMO patients dying compared with 1 of 7 VV-ECMO patients.	High. Small subgroups and likely confounding by indication.
Sugiyama et al., 2019	Single-center retrospective cohort.	35 PEA patients, 4 VA-ECMO cases.	All 4 patients were weaned from VA-ECMO and 3 of 4 survived to discharge.	High. Very small cohort, so estimates are imprecise.
Grate et al., 2025	Case report.	1 patient on VV-ECMO.	Bacteremia caused purulent thrombosis of the membrane oxygenator. Treated with circuit exchange, antibiotics, and PTE.	High. Single case report.
Long et al., 2021	Case report.	1 patient on VA-ECMO after PEA.	Developed secondary LV dysfunction during VA-ECMO. Weaned on POD 7, but later had brain ischemia and was transferred to a local hospital on POD 16.	High. Single case report.
Nakamura et al., 2015	Case report.	1 patient on VA-ECMO after PEA.	Rescue BPA allowed VA-ECMO to be removed 5 days later. The patient was discharged after 139 days.	High. Single case report.

## Data Availability

No new data were created or analyzed in this study.

## Abbreviations

BMI	Body Mass Index
BNP	B-type Natriuretic Peptide
BPA	Balloon Pulmonary Angioplasty
CPB	Cardiopulmonary Bypass
CRP	C-reactive protein
CTEPH	Chronic Thrombo-Embolic Pulmonary Hypertension
CTPA	Computed Tomography Pulmonary Angiography
DRHF	Decompensated Right Heart Failure
ECMO	Extracorporeal Membrane Oxygenation
MCS	Mechanical Circulatory Support
mPAP	Mean Pulmonary Arterial Pressure
N/L	Neutrophil-to-Lymphocyte ratio
PAH	Pulmonary Arterial Hypertension
PAWP	Pulmonary Artery Wedge Pressure
PEA	Pulmonary Endarterectomy
PVR	Pulmonary Vascular Resistance
TR	Tricuspid Regurgitation
V-AV	Veno-Arteriovenous
VA ECMO	Veno-arterial extracorporeal membrane oxygenation
V-VA	Veno-venoarterial
VPMD	Ventilation-perfusion Mismatch Defect
VV ECMO	Veno-venous extracorporeal membrane oxygenation
